# The SARS-CoV-2 inactivated vaccine enhances the broad neutralization against variants in individuals recovered from COVID-19 up to one year

**DOI:** 10.1080/22221751.2022.2043728

**Published:** 2022-03-03

**Authors:** Zheng Zhang, Bin Ju, Xinrong Zhou, Lin Cheng, Haiyan Wang, Xuejiao Liao, Miao Wang, Lanlan Wei, Shuo Song, Bing Zhou, Zhenghua Ma, Huimin Guo, Xiangyang Ge

**Affiliations:** a Institute for Hepatology, National Clinical Research Center for Infectious Disease, Shenzhen Third People’s Hospital; The Second Affiliated Hospital, School of Medicine, Southern University of Science and Technology, Shenzhen, People’s Republic of China; b Follow-up Department of Chronic Diseases, National Clinical Research Center for Infectious Disease, Shenzhen Third People’s Hospital; The Second Affiliated Hospital, School of Medicine, Southern University of Science and Technology, Shenzhen, People’s Republic of China; c Guangdong Key Laboratory for Anti-Infection Drug Quality Evaluation, Shenzhen, People’s Republic of China; d Shenzhen Research Center for Communicable Disease Diagnosis and Treatment of Chinese Academy of Medical Science, Shenzhen, People’s Republic of China

Since the outbreak of the coronavirus disease 2019 (COVID-19) in December 2019 [1], the continuously emerging severe acute respiratory syndrome coronavirus 2 (SARS-CoV-2) variants have been identified and reported in different regions and countries worldwide, such as Alpha (B.1.1.7), Beta (B.1.351), Gamma (P.1), Delta (B.1.617.2), Omicron (B.1.1.529), Kappa (B.1.617.1), Eta (B.1.525), and Iota (B.1.526) [2,3]. SARS-CoV-2 variants have contributed to several waves of the COVID-19 pandemic, whose transmissibility and infectivity are much higher than the original wild-type (WT) virus. More seriously, these variants largely escaped the neutralization by convalescent and vaccine-elicited plasma and monoclonal neutralizing antibodies (nAbs), greatly hindering the development of effective measures to prevent and control the virus infection.

Vaccination has long been a crucial measure to protect human against the infection of pathogens and can establish solid immune barriers in populations. Currently, various kinds of SARS-CoV-2 vaccines including inactivated vaccine (Sinopharm, Sinovac), DNA vaccine (Inovio), mRNA vaccine (Pfizer/BioNTech, Moderna), adenovirus vector vaccine (AstraZeneca/Oxford, Johnson & Johnson, Cansino), and protein vaccine (Novavax, Zhifei), showed good efficacy and therefore were adopted by various countries for population immunization [4–7]. For the individuals previously infected with SARS-CoV-2, it is debated whether they should be vaccinated or not. Indeed, the natural virus infection could induce robust antibody responses in COVID-19 patients which could be maintained after 7 months since the symptom onset [8]. However, the neutralizing activities of the convalescent plasma were gradually decreased, especially after one year of recovery [9], suggesting a risk of re-infection of SARS-CoV-2 variants.

Indeed, several studies have reported that breakthrough infections occurred in some vaccine recipients, indicating that there is a strong correlation between the immune protection and the value of nAbs [10]. Therefore, it is very important to monitor the longitudinal dynamics of plasma nAbs against the emerging SARS-CoV-2 variants continuously. The antibody responses to the mRNA and viral vector vaccines have been evaluated in individuals who previously infected with SARS-CoV-2 [11]. It is found that the high levels of nAbs against both the WT virus and variants were initialized by one or two doses of vaccines [12]. However, the antibody response to SARS-CoV-2 variants boosted by the inactivated vaccine in convalescent individuals is still unknown and the level of enhancement and the broad spectrum in neutralizing activity remain elusive.

In this study, we monitored the longitudinal dynamics of plasma IgG, IgA, and IgM binding to the SARS-CoV-2 WT receptor binding domain (RBD) in 22 of convalescent COVID-19 individuals who received at least one dose of inactivated vaccine (Figure S1A, Table S1). The levels of RBD-specific antibodies gradually declined with time over. Then we evaluated the changes of plasma antibodies after the inoculation of the inactivated vaccine. The follow-up period was divided into three phases including the early stage of recovery (median time: one month), late stage of recovery (median time: one year, i.e. before vaccination), and post vaccination (Figure S1B). The geometric mean values of RBD-specific IgG, IgA, and IgM decreased to 23.7%, 34.4%, and 29.1%, respectively in the late stage of recovery (Figure S1C). After the vaccination with inactivated vaccine, the levels of IgG had 3.9-fold increase as compared with those before vaccination, and reached the similar levels with those in the early stage of recovery. By contrast, the boosted vaccination failed to induce a recalling IgA or IgM response, suggesting that virus-specific IgG may play a more important role in the long-term antibody protection.

To further evaluate the neutralizing activities of plasma against SARS-CoV-2 variants, we constructed seven kinds of pseudoviruses based on the HIV-1 backbone, including WT (Wuhan reference strain), Alpha, Beta, Delta, Kappa, Eta, and Iota variants (Figure S2), and then performed the pseudovirus-neutralization assay. The diverse mutations in the region of spike protein contributed to their distinct neutralizing resistance. As shown in Figure 1(a), Figure S3, and Table S2, although the convalescent plasma showed effective neutralizing activities in the early stage of recovery, their geometric mean titers (GMTs) of nAbs were largely decreased in the late stage of recovery, especially against various SARS-CoV-2 variants. After the boosted vaccination with inactivated vaccine, the neutralizing activities of plasma against the WT and six mutated viruses we tested were significantly increased 6.9-fold to 17.3-fold as compared with those before vaccination, whose levels were compatible to those in the early stage of recovery.

**Figure 1.**
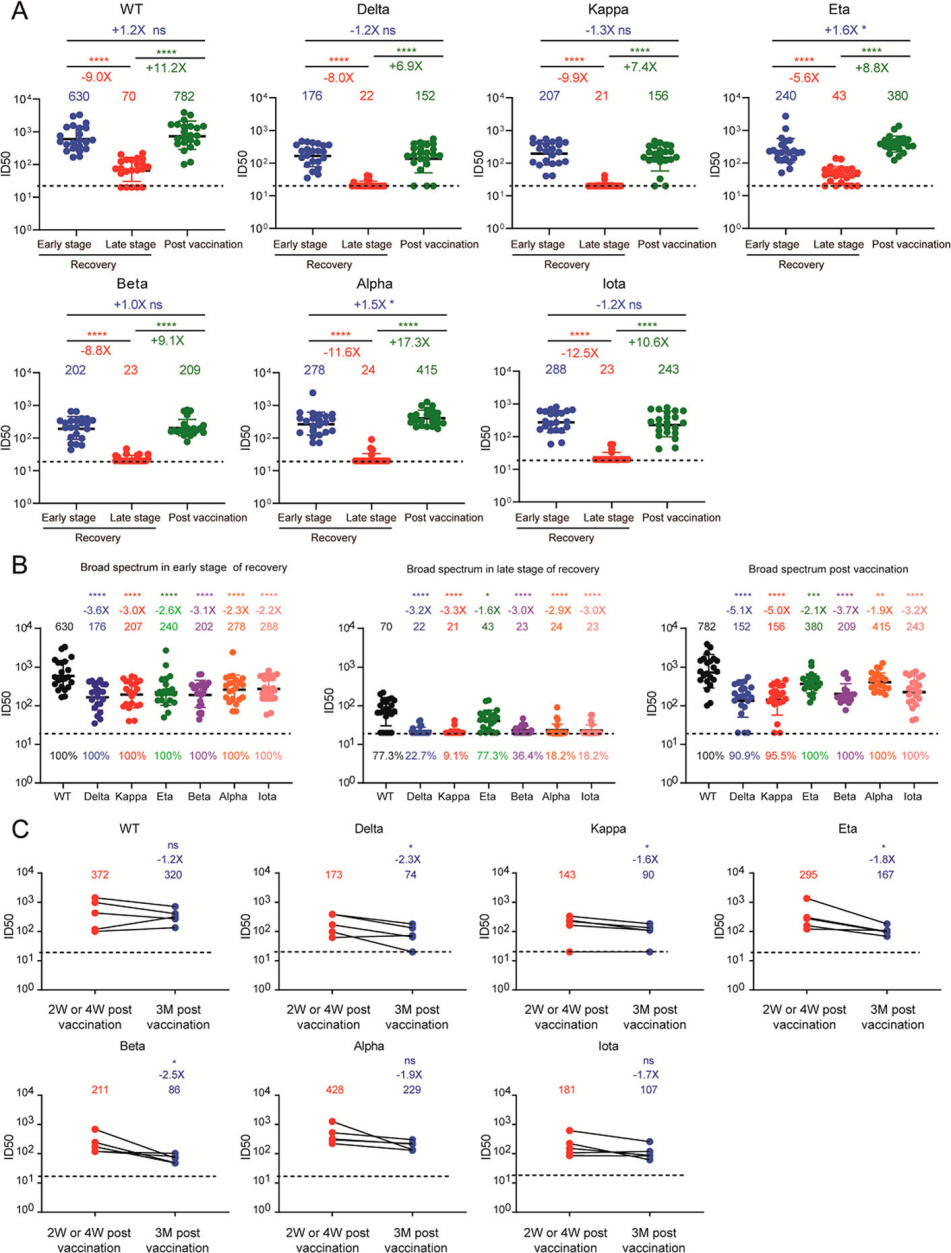
Neutralizing activities against WT SARS-CoV-2 and variants in convalescent individuals received the inactivated vaccine. (a) Plasma neutralizing activities against each SARS-CoV-2 strain in three follow-up time points were measured and shown in the values of 50% inhibition dilution (ID_50_). (b) The broad spectrum of plasma nAbs in three follow-up time points. The GMT of nAbs against each variant was compared to that against WT, respectively. The positive rates of nAbs were marked at the bottom of each column. (c) The durability of broadly nAbs post the boosted vaccination. Paired plasma samples were collected from five individuals at Week 2 (*n* = 4) or Week 4 (*n* = 1) and Month 3 post vaccination and then tested the neutralizing activities against WT SARS-CoV-2 and variants. “-” represents decreased neutralization activity, “+” represents increased neutralization activity. The paired *t* test is performed here. “****” means *P* < .0001, “***” means *P* < .001, “**” means *P* < .01, “*” means *P* < .05, “ns” means not significant. The GMT, fold-change, and significance of difference were labeled on the top. The limit of detection was 1:20 dilution. The data below the limit was set to 20 for visualization.

To better evaluate the broadness of plasma nAbs in different stages of follow-up, we rearranged these neutralization results by different time periods to directly compare their neutralizing values against SARS-CoV-2 variants. As shown in Figure 1(b), the plasma collected in the early stage of recovery had high levels of nAbs against both WT SARS-CoV-2 and variants, whose GMTs ranged from 176 to 630 and neutralizing antibody positive rates were 100% in all seven tested viruses. Along with the time over, the levels of nAbs were significantly decreased after one year. Especially, most of plasma samples lost their neutralizing activities against SARS-CoV-2 variants, and the positive rates of nAbs had also been dropped to 9.1% to 77.3%. Among them, a total of 22 individuals accepted at least one dose of inactivated vaccine and contributed their blood samples. The levels of nAbs were remarkably increased as compared with those before vaccination and rapidly raised to the similar levels in the early stage of recovery. Meanwhile, the positive rates of nAbs against SARS-CoV-2 variants were also increased to 90.9% to 100%. Thus, we clearly demonstrated that the vaccination with inactivated vaccine rapidly enhanced the neutralizing activities against the SARS-CoV-2 variants in individuals who have recovered from COVID-19 up to one year.

Finally, we also explored the durability of neutralizing antibody response elicited by the boosted vaccination in these convalescent individuals. We have obtained serial plasma samples from five individuals (donor 2, 11, 16, 19 and 20) at Week 2 or Week 4 and Month 3 post vaccination. As shown in Figure 1(c) and Figure S4, the levels of nAbs were slightly decreased with time, but dropped more obviously against the variants including Delta, Kappa, Eta, and Beta. These results suggested that it is very important to monitor the levels of nAbs against the emerging SARS-CoV-2 variants in the convalescent COVID-19 individuals.

Compared with several previous studies, the results described here were rational and novel. Xiang et al detected the levels of nAbs against Beta variant in convalescent patients one year after infection, and found that those individuals who effectively neutralized the WT virus displayed limited neutralizing activities against Beta variant (diminished to 22.6%) [13]. Furthermore, Li et al detected the RBD-specific antibody responses in 1782 plasma samples from 869 convalescent donors after 12 months post infection in Wuhan, China, and found that the levels of plasma IgG and nAbs significantly declined with time [9]. Combined with our study, these results emphasized the risk of reinfection with SARS-CoV-2 variants in convalescent COVID-19 individuals recovered more than one year. Since the different vaccines have diverse immunogenicity, the effectiveness of all being applied SARS-CoV-2 vaccines should be comprehensively evaluated. Lucas et al had analyzed the immune response to SARS-CoV-2 in the cohorts of previously infected (recovered) or uninfected (naive) individuals who received the mRNA vaccine. The results showed that individuals in both groups obtained neutralization capacity against all tested variants. Moreover, plasma samples from previously infected individuals exhibited better neutralizing activities than those from uninfected donors generally. After the vaccination with mRNA vaccine, the high levels of nAbs could persist about 4–6 months, and were then reduced over time because of the waning immunity [14]. In addition, similar immune responses were also observed in the population who previously infected with SARS-CoV-2 and then received one dose of Ad26.COV2.S vaccine or the replicating poxvirus vector-based RBD vaccine, suggesting that the boosted vaccination could bring a solid immune protection to the convalescent individuals. However, it lacks the report about the antibody responses in convalescent individuals after the boosted vaccination with the inactivated vaccine. Our results here demonstrated that the vaccination with inactivated vaccine was also effective in enhancing the levels of nAbs in convalescent individuals, especially against the emerging SARS-CoV-2 variants. Importantly, according to a recent research report, the titers of nAbs were positive correlation with immune efficacy against COVID-19. The vaccinators with ID_50_ values of 10, 100, and 1000 owned 78%, 91%, and 96% immune efficacy, respectively, after 4 weeks inoculated with mRNA vaccine [15], suggesting that the convalescent patients obtained high immune efficacy in the early stage of recovery and post the vaccination with inactivated vaccine in this study.

In conclusion, we evaluated the plasma neutralization against various SARS-CoV-2 variants in the convalescent individuals who received the inactivated vaccine. These results showed that the levels of broadly nAbs were significantly decreased in the convalescent individuals after one year since they recovered from COVID-19, suggesting the high risk of reinfection of various emerging variants. The vaccination with inactivated vaccine potentially improved the plasma neutralizing activities against the WT SARS-CoV-2 and variants, which could even last for 3 months post vaccination. This study for the first time demonstrated that the inactivated vaccine potentially induced the neutralizing activity against the emerging SARS-CoV-2 variants in the convalescent individuals, which could minimize the risk of breakthrough infections in future.

## Supplementary Material

Supplemental Material

## Data Availability

We are happy to share reagents and information in this study upon request.
