# Supplementary material for: The SARS-CoV-2 inactivated vaccine enhances the broad neutralization against variants in individuals recovered from COVID-19 up to one year
Source: Emerg Microbes Infect. 2022 Mar 3;11(1):753–6. doi: 10.1080/22221751.2022.2043728 (PMC8903755; doi:10.1080/22221751.2022.2043728)
Supplement: Supplemental Material [file TEMI_A_2043728_SM6132.zip › Suppl files/Patient_Vac_supplementary_information_20220206_clean copy.docx]

**Supplementary information**

**Materials and Methods**

**Study approval and plasma samples**

This study was approved by the Ethics Committee of Shenzhen Third People’s Hospital, China. All participants had provided written informed consent for sample collection and subsequent analysis. All convalescent individuals were administrated with at least one dose of SARS-CoV-2 inactivated vaccine (Sinovac: 13 donors, Sinopharm: 8 donors, and Shenzhen Kangtai: 1 donor). Plasma samples were collected and stored at -80 °C in the Biobank of the Shenzhen Third People’s Hospital, and heat-inactivated at 56 °C for 30 mins before use. Plasma is suitable for the antibody detection and usually used in the binding and neutralizing assay^1,2^. The values of SARS-CoV-2 RBD-specific plasma IgG, IgA, and IgM were measured using the Chemiluminescence immunoassay kit as previous studies^3-5^. These values were shown in cut-off index (COI) and obtained from the routine clinical detection item.

**SARS-CoV-2 pseudovirus-based neutralizing assay**

The SARS-CoV-2 wild-type and mutated pseudoviruses were generated by co-transfection of HEK-293T cells with 10 μg of spike-expressing plasmid and 20 μg of the env-deficient HIV-1 backbone vector (pNL4-3.Luc.R-E-)^3^. Two days post-transfection, the culture supernatant was harvested, clarified by centrifugation, filtered, and stored at -80 °C. To determine the neutralizing activity, 5-fold serially diluted plasma samples (initial dilution 1:20) were incubated with the equal volume of diluted pseudovirus at 37 °C for 1 h. The HEK-293T-hACE2 cells were subsequently added in duplicate. After a 48 h incubation, the culture medium was removed and 100 μL of the Bright-Lite Luciferase reagent (Vazyme Biotech) was added to the cells. After a 2-min incubation at RT, 90 μL of cell lysate was transferred to the 96-well white solid plates for measuring the luminescence using the Varioskan™ LUX multimode microplate reader (Thermo Fisher Scientific). The 50% inhibitory dilution (ID_50_) was calculated using GraphPad Prism 8.0 software by log (inhibitor) vs. normalized response - Variable slope (four parameters) model.

**Statistical analysis**

Statistical analysis was performed with unpaired or paired *t* test using GraphPad Prism 8.0 software. ‘*’ means P < 0.05, ‘**’ means P < 0.01, ‘***’ means P < 0.001, ‘****’ means P < 0.0001, “ns” means not significant.

**Reference**

1. Ai J, Zhang H, Zhang Y, et al. Omicron variant showed lower neutralizing sensitivity than other SARS-CoV-2 variants to immune sera elicited by vaccines after boost. Emerg Microbes Infect 2021;1-24.

2. Zhang X, Wu S, Wu B, et al. SARS-CoV-2 Omicron strain exhibits potent capabilities for immune evasion and viral entrance. Signal Transduct Target Ther 2021;6:430.

3. ZhaoJ, Yuan Q, Wang H, et al. Antibody Responses to SARS-CoV-2 in Patients With Novel Coronavirus Disease 2019. Clin Infect Dis 2020;71,:2027-2034.

4. Yu S, An J, Liao X, et al. Distinct kinetics of immunoglobulin isotypes reveal early diagnosis and disease severity of COVID-19: A 6-month follow-up. Clin Transl Med 2021;11: e342.

5. Cheng L, Song S, Fan Q, et al. Cross-neutralization of SARS-CoV-2 Kappa and Delta variants by inactivated vaccine-elicited serum and monoclonal antibodies. Cell Discov 2021; 7:112.


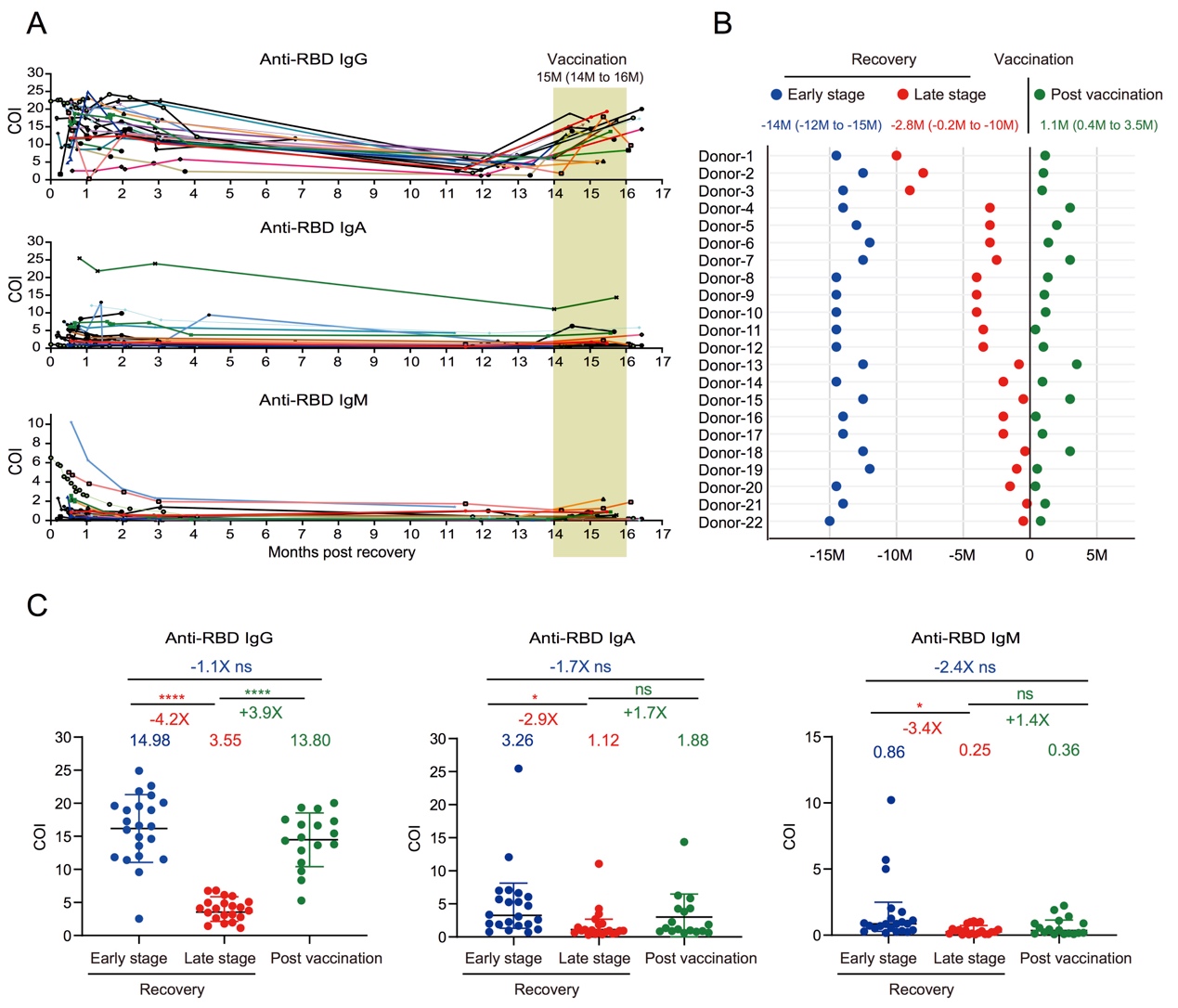


**Supplementary Figure S1. Longitudinal** **dynamics of plasma IgG, IgA, and IgM binding to the SARS-CoV-2 WT RBD.**

**(A)** Dynamics of RBD-specific plasma IgG, IgA, and IgM in 22 convalescent individuals at different follow-up time points. First dose of inactivated vaccine administration was conducted post more than one year after recovery (Median time: 15 months, ranged from 14 to 16 months). The value of plasma antibody was shown in cut-off index (COI). Each color of line represents one donor. **(B)** Schematic diagram of samples collection. The follow-up was divided into three periods including early stage of recovery, late stage of recovery, and post vaccination. The date of vaccination was normalized as Day 0 and the median time of each stage was labeled on the top. **(C)** Comparative analysis of plasma IgG, IgA, and IgM levels before vaccination (early stage and late stage of recovery) and post boosted vaccination with inactivated vaccine. The number of donors in early stage of recovery was 22, in late stage was 21, and post vaccination was 17. Symbol “-” represents decreased antibody value, “+” represents increased antibody value. The unpaired *t* test is performed here. “****” means P < 0.0001, “*” means P < 0.05, “ns” means not significant.


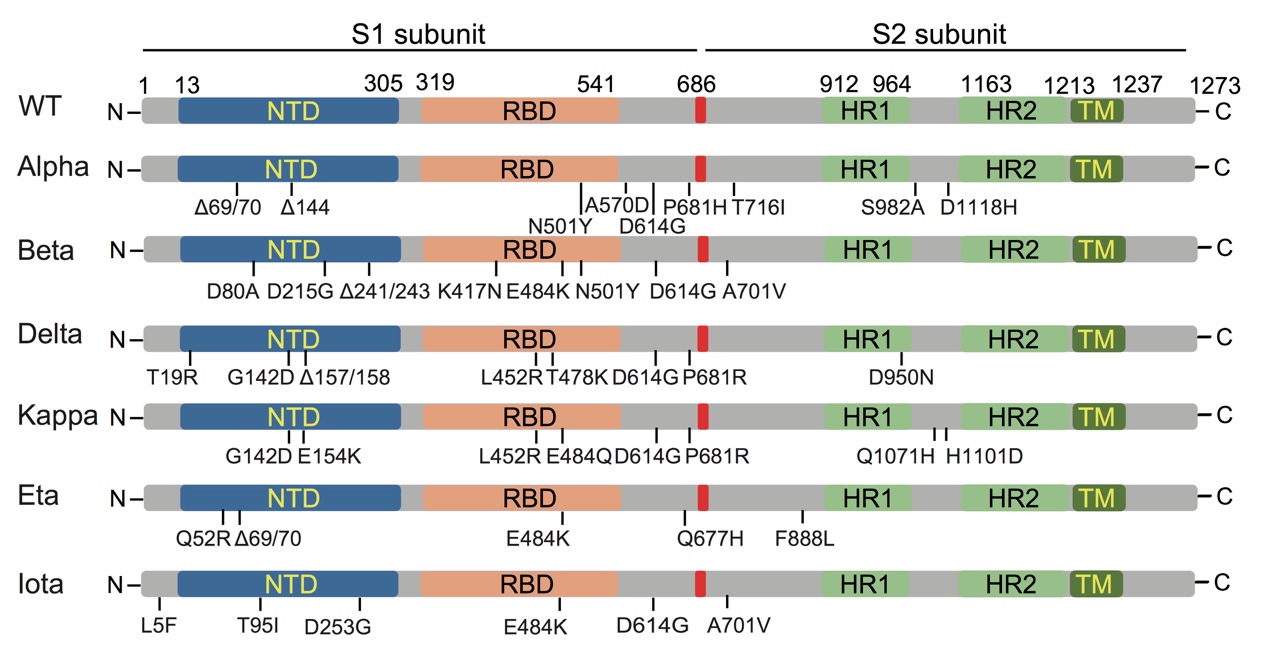


**Supplementary Figure S2. Mutations in the spike protein of SARS-CoV-2 variants compared to the WT strain.**

Total 7 kinds of SARS-CoV-2 pseudoviruses, including the Wuhan reference strain and 6 variants, were used to analyze the neutralizing activity of plasma.


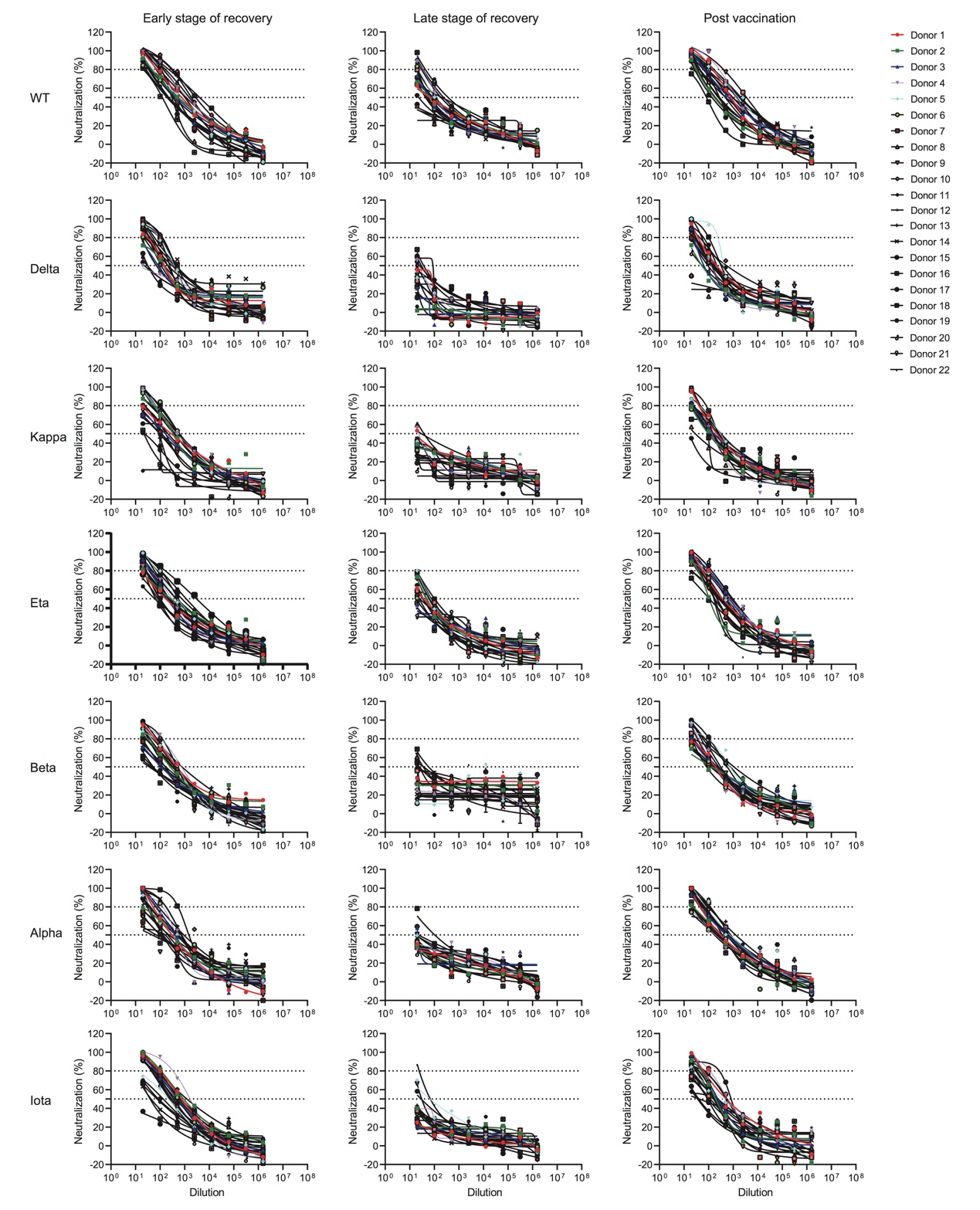


**Supplementary Figure S3. The neutralization of plasma sample of 22 individuals at three follow-up time points against the WT SARS-CoV-2 and variants.**

Plasma samples of 22 individuals at three follow-up time points were tested. One representative curve from two to four independent experiments was displayed.


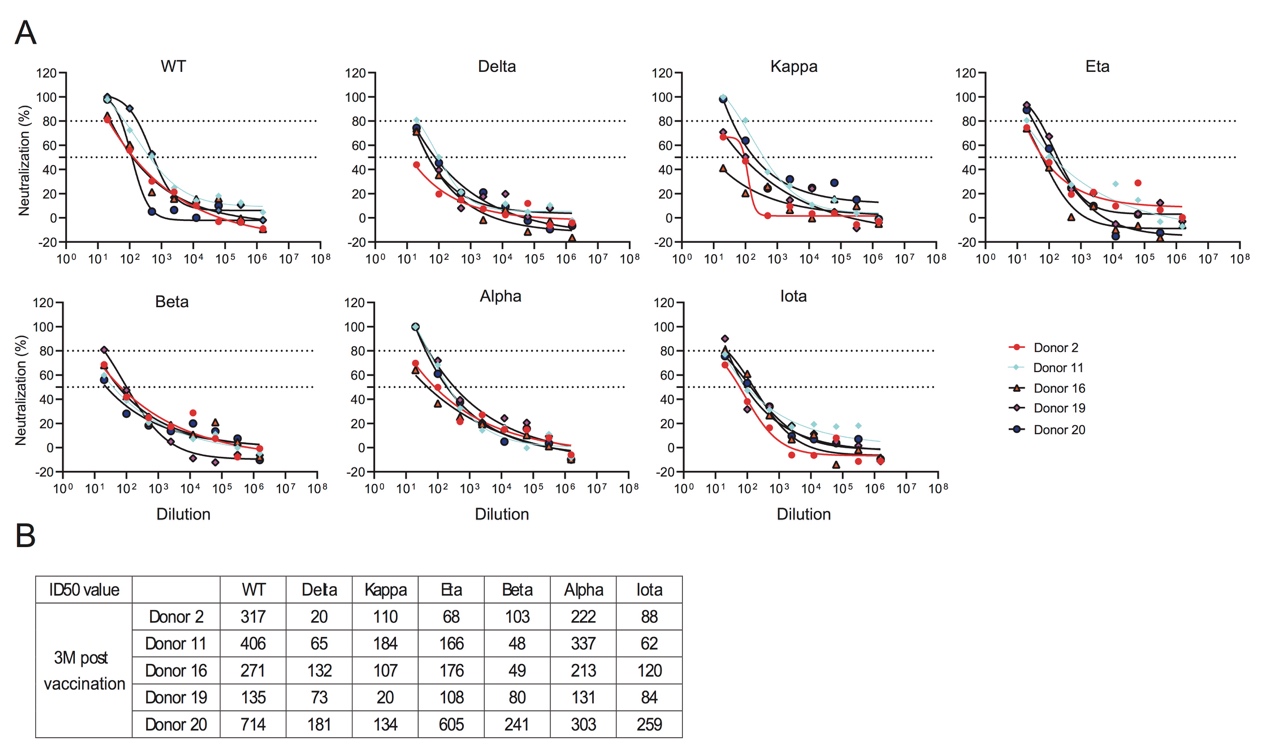


**Supplementary Figure S4. The neutralization of plasma sample of 5 individuals at three-month post vaccination.**

**(A)** Plasma samples of 5 individuals at Month 3 post vaccination were tested. One representative curve from two to four independent experiments was displayed. **(B)** The neutralization of each plasma was displayed in ID_50_. The data was shown in mean of two to four independent experiments. The data below the limit of detection (1:20) was set to 20 for visualization.

**Supplementary Table S1. The information of study subjects and the time points of vaccination and blood sampling.**


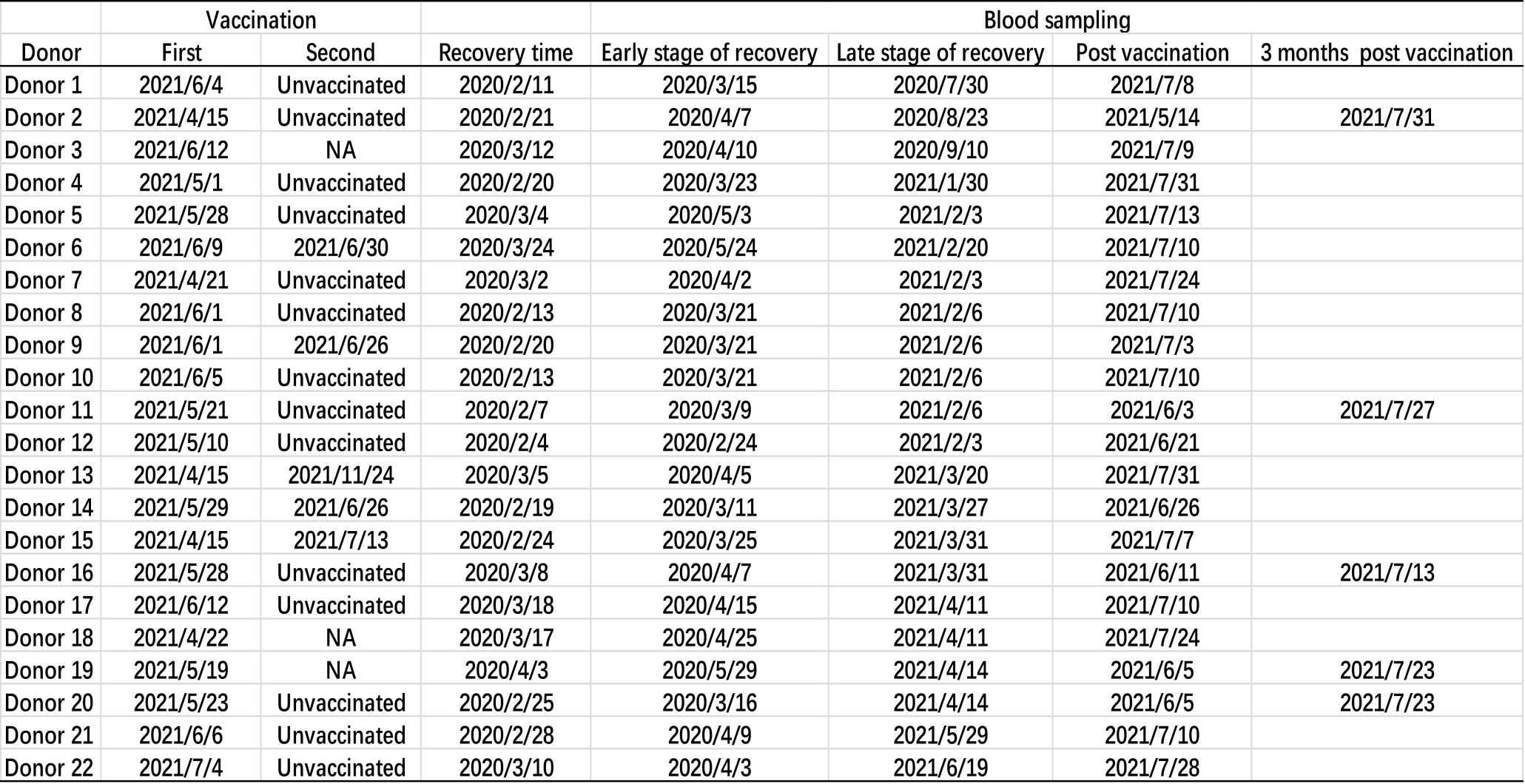


n.a.: not available.

**Supplementary Table S2. The ID_50_ values of each plasma from 22 individuals at three follow-up time points against 7 SARS-CoV-2 strains were calculated and listed.**


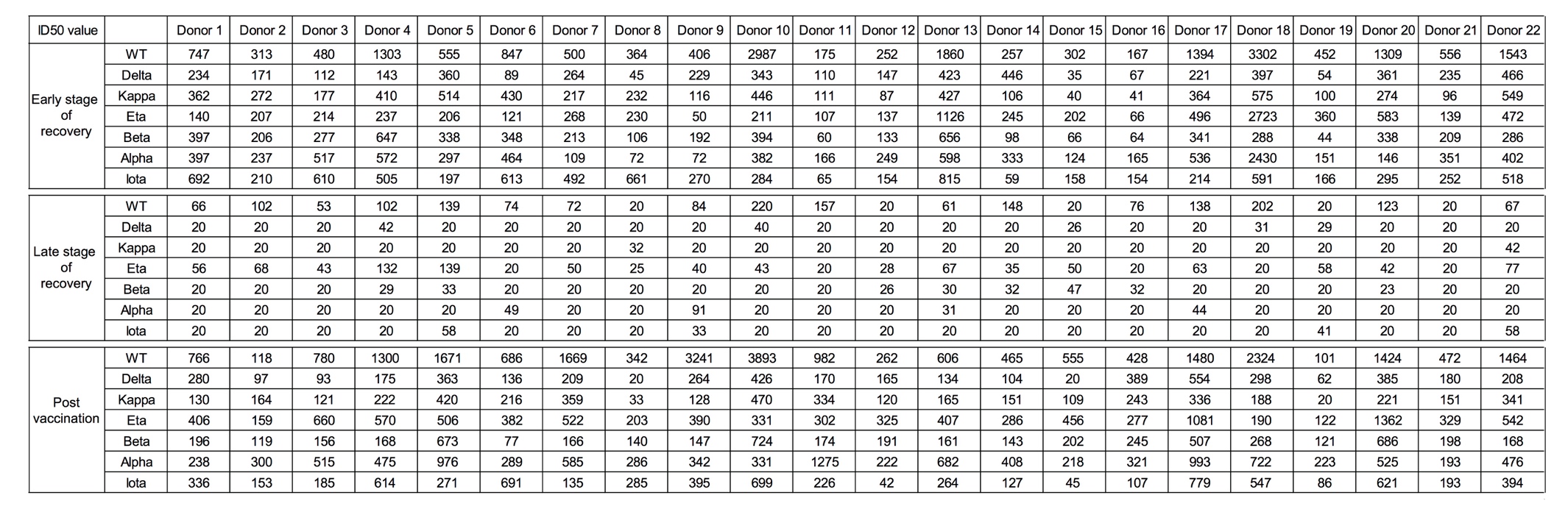


The data was shown in mean of two to four independent experiments. The data below the limit of detection (1:20) was set to 20 for visualization.
