# Supplementary figures and images for: The SARS-CoV-2 inactivated vaccine enhances the broad neutralization against variants in individuals recovered from COVID-19 up to one year
Source: Emerg Microbes Infect. 2022 Mar 3;11(1):753–6. doi: 10.1080/22221751.2022.2043728 (PMC8903755; doi:10.1080/22221751.2022.2043728)

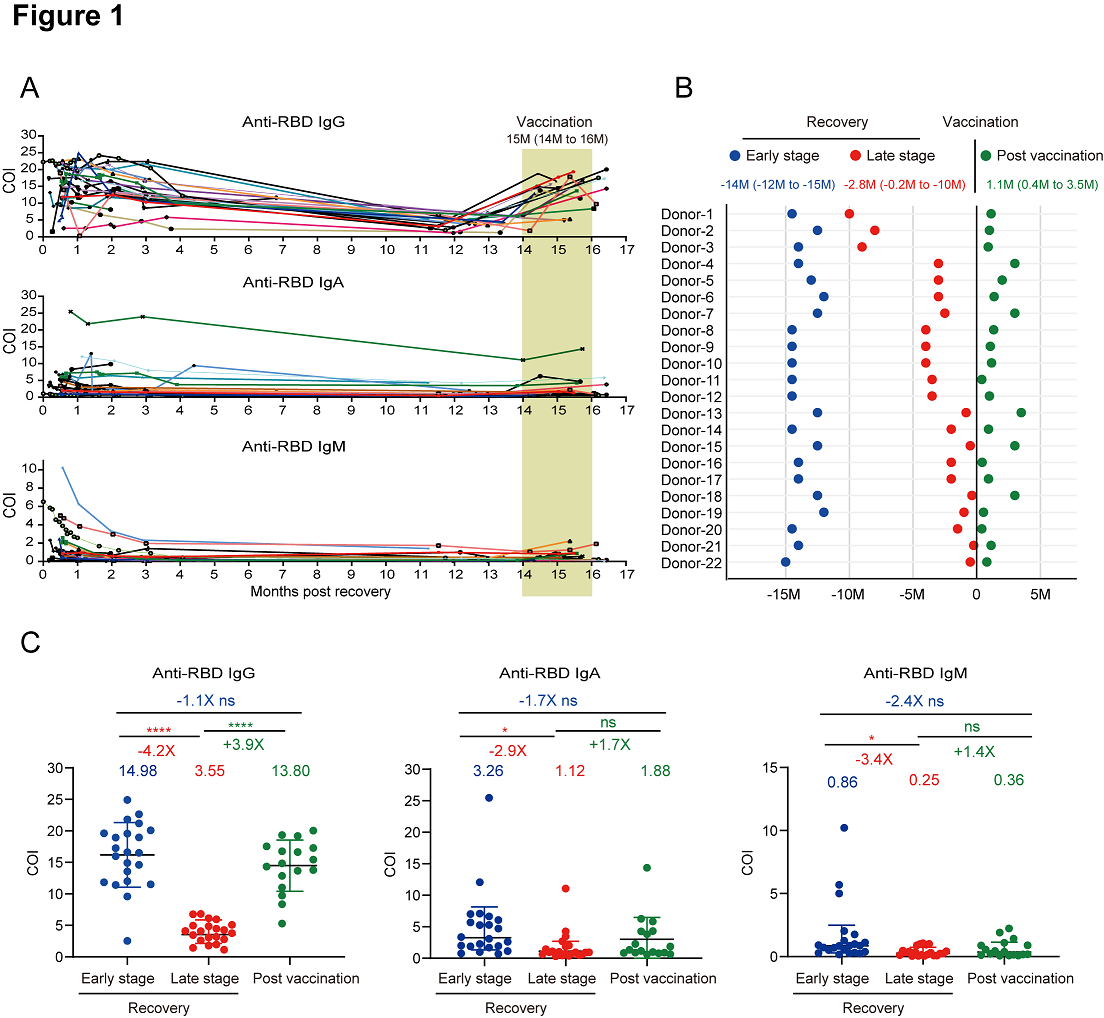

Supplement: Supplemental Material [file TEMI_A_2043728_SM6132.zip › Suppl files/Supplementary_Figure_S1_Longitudinal_dynamics_of_plasma_IgG_IgA_and_IgM_binding_to_the_SARSCoV2_WT_RBD_.png]

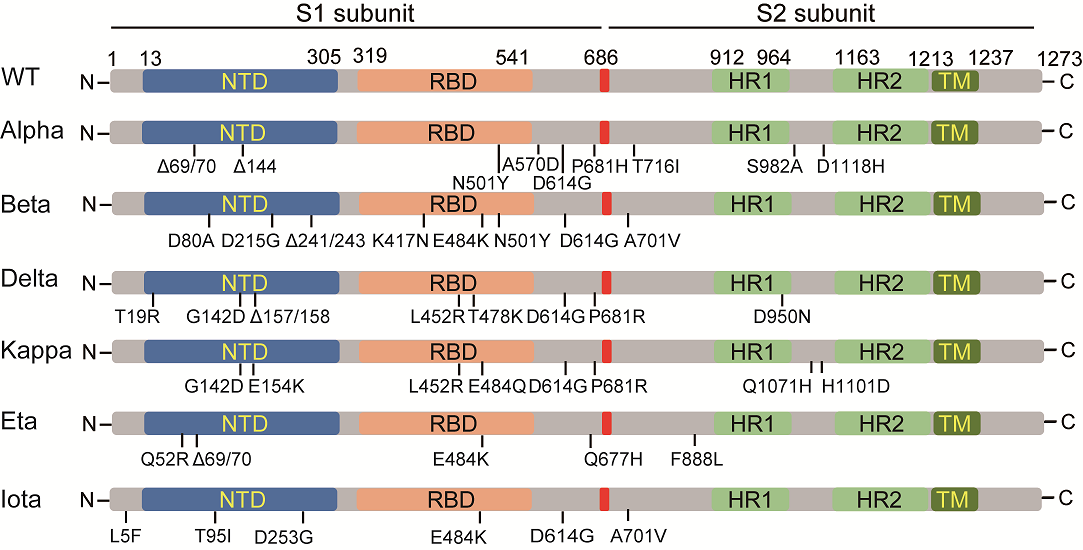

Supplement: Supplemental Material [file TEMI_A_2043728_SM6132.zip › Suppl files/Supplementary_Figure_S2_Mutations_in_the_spike_protein_of_SARSCoV2_variants_compared_to_the_WT_strain_.png]

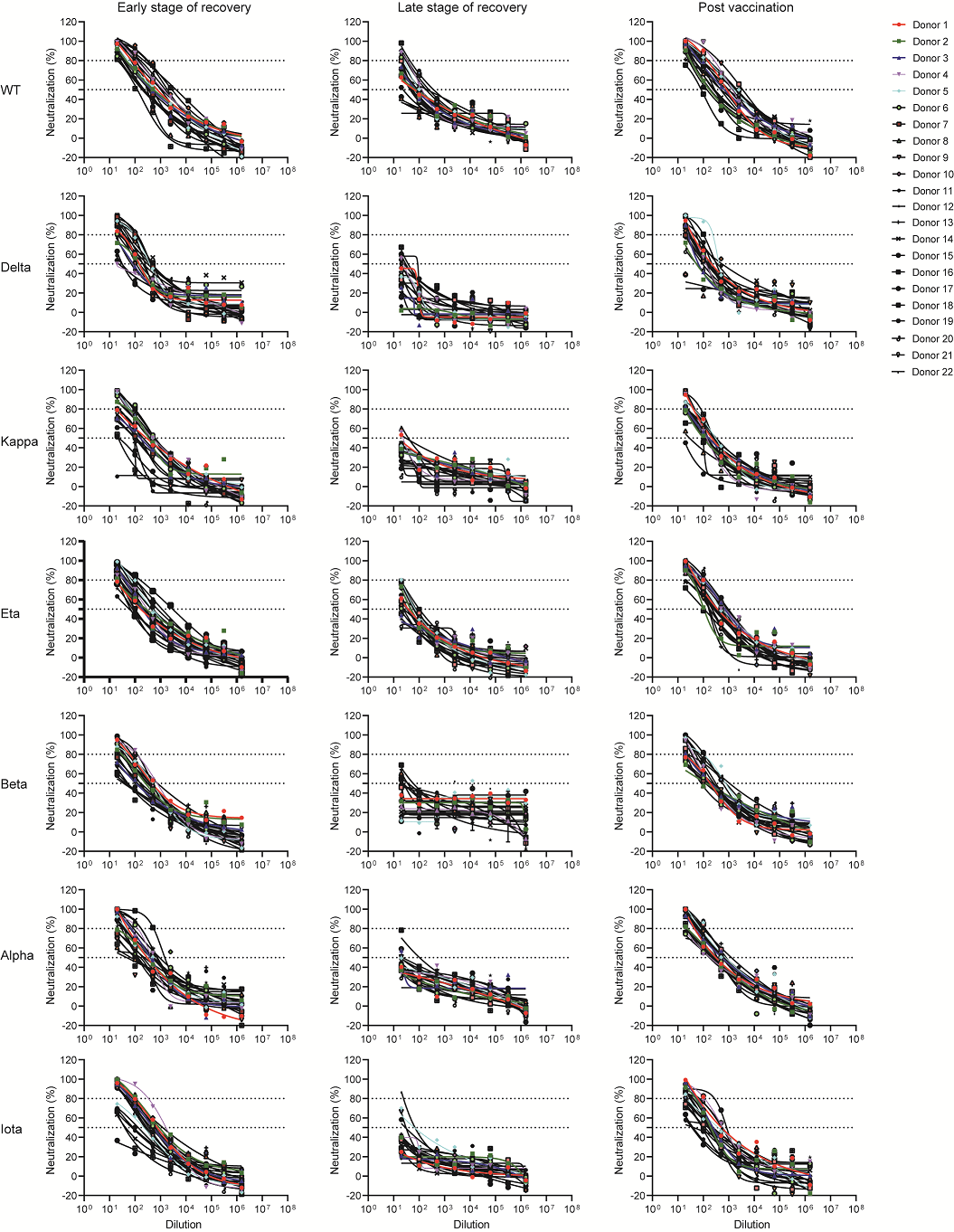

Supplement: Supplemental Material [file TEMI_A_2043728_SM6132.zip › Suppl files/Supplementary_Figure_S3_The_neutralization_of_plasma_sample_of_22_individuals_at_three_follow_up_time_points_against_the_WT_SARSCoV2_and_variants_.png]

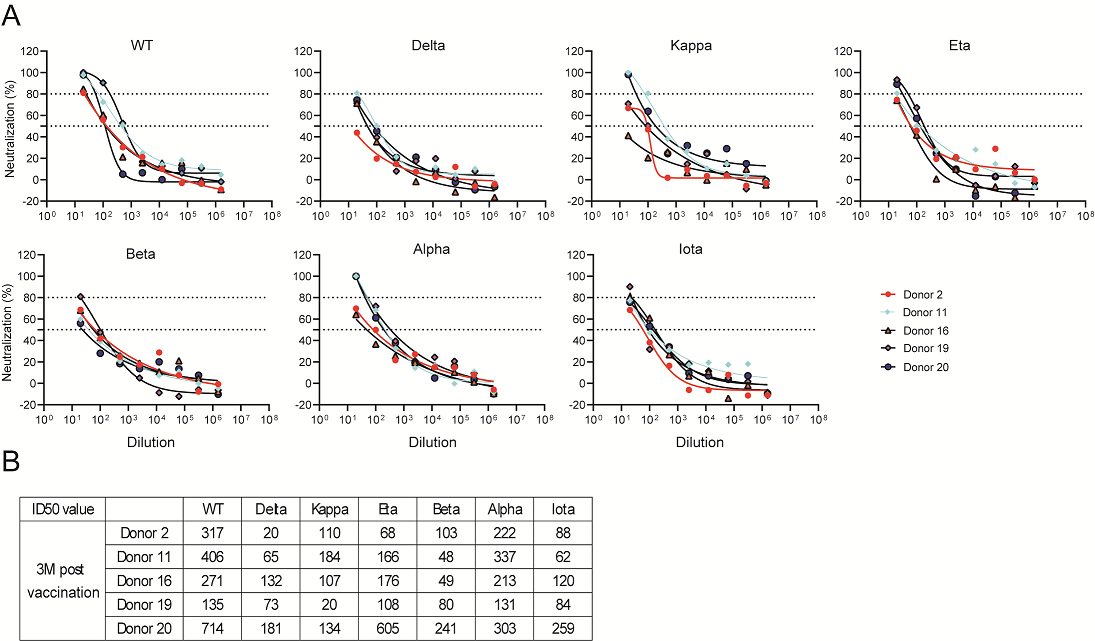

Supplement: Supplemental Material [file TEMI_A_2043728_SM6132.zip › Suppl files/Supplementary_Figure_S4_The_neutralization_of_plasma_sample_of_5_individuals_at_three_month_post_vaccination_.png]
